# Obituaries

**Published:** 1912-11

**Authors:** 


In the 33 deaths of physicians chronicled in the Journal of the A. M. A., Sept. 28, nine were of Civil War veterans. Aside from the historic interest and the heroic association connected with these men, which should not fail to impress the rising generation of physicians, we are at a period of professional change which will probably never be duplicated, and which is of interest in many ways, economic among others. The comparatively sparse settlement of the country in the middle decades of the last century, the occurrence of the Civil War and the development of the West immediately following, in connection with the necessarily low educational requirements of the times, and the incentives to establishing medical schools, led to the graduation of a large number of men and, especially of men past the first flush of youth. It is probable that the medical profession, at present, has a greater proportion of old and retired members than it will have in the future and that by the inevitable high death rate of this part of its membership, and the restrictions on medical education now in force, the next decade will see an absolute as well as a relative decline in total numbers.

